# Vortex dynamics in NbTi films at high frequency and high DC magnetic fields

**DOI:** 10.1038/s41598-023-36473-x

**Published:** 2023-06-08

**Authors:** Gianluca Ghigo, Daniele Torsello, Laura Gozzelino, Michela Fracasso, Mattia Bartoli, Cristian Pira, Davide Ford, Giovanni Marconato, Matteo Fretto, Ivan De Carlo, Nicola Pompeo, Enrico Silva

**Affiliations:** 1 Department of Applied Science and Technology, Politecnico di Torino, 10129 Turin, Italy; 2 Istituto Nazionale di Fisica Nucleare, Sezione di Torino, 10125 Turin, Italy; 3 Center for Sustainable Future Technologies, Italian Institute of Technology, 10144 Turin, Italy; 4 Consorzio Interuniversitario Nazionale per la Scienza e Tecnologia dei Materiali (INSTM), 50121 Florence, Italy; 5 Laboratori Nazionali di Legnaro, Istituto Nazionale di Fisica Nucleare, 35020 Legnaro, Italy; 6 Istituto Nazionale di Ricerca Metrologica, 10135 Turin, Italy; 7 Department of Electronics and Telecommunications, Politecnico di Torino, 10129 Turin, Italy; 8 Department of Industrial, Electronic and Mechanical Engineering, Università Roma Tre, 00146 Rome, Italy; 9 Istituto Nazionale di Fisica Nucleare, Sezione di Roma Tre, 00146 Rome, Italy

**Keywords:** Condensed-matter physics, Superconducting properties and materials

## Abstract

We report on the characterization of NbTi films at $\sim$ 11 GHz and in DC magnetic fields up to 4 T, performed by means of the coplanar waveguide resonator technique, providing quantitative information about the penetration depth, the complex impedance, and the vortex-motion-induced complex resistivity. This kind of characterization is essential for the development of radiofrequency cavity technology. To access the vortex-pinning parameters, the complex impedance was analyzed within the formalism of the Campbell penetration depth. Measurements in this frequency range allowed us to determine the complete set of vortex-pinning parameters and the flux flow resistivity, both analyzed and discussed in the framework of high-frequency vortex dynamics models. The analysis also benefits from the comparison with results obtained by a dielectric-loaded resonator technique on similar samples and by other ancillary structural and electromagnetic characterization techniques that provide us with a comprehensive picture of the material. It turns out that the normalized flux flow resistivity follows remarkably well the trend predicted by the time dependent Ginzburg-Landau theory, while the pinning constant exhibits a decreasing trend with the field which points to a collective pinning regime.

## Introduction

The NbTi alloy is widely used in superconducting applications, especially for magnets^1^, thanks to its desirable metallurgical properties. This explains why this compound has been studied deeply in the form of filaments and wires, with the goal to optimize its current carrying capability and critical field. Another application field where Nb-based materials plays a role is as coating for superconducting radiofrequency cavities, to be used in particle accelerators^2^. Recently, there has been a renewed interest for radiofrequency cavities due to their employment as axion haloscopes to the search for dark matter^3,4^. In this case, cavities are exposed to magnetic fields of several tesla, since theoretical models predict that passing through a magnetic field axions will be converted to photons with frequency related to their mass^5^. Those matching the cavity resonance frequency should produce a measurable signal. This example highlights the need for a comprehensive understanding of the basic mechanisms of dissipation in superconducting films at high frequency and in high DC magnetic fields, extreme conditions that have not been explored extensively in the past.

In this work, we report on a complete characterization of Nb$_{0.31}$Ti$_{0.69}$ films at $\sim 11$ GHz and in DC magnetic fields up to 4 T. Under these conditions, the superconductor is in the vortex state, with substantial amounts of fluxons whose dynamics is expected to be the dominant source of surface resistance. We adopted the coplanar waveguide resonator (CPWR) technique that, through the analysis of the behavior of a resonator made by patterning the film to be studied, provides quantitative information about the penetration depth, the complex impedance, and the vortex-motion-induced complex resistivity. In fact, measurements in this frequency range allow determining both the vortex pinning properties and the flux flow resistivity, that is analyzed and discussed in the framework of high-frequency vortex dynamics models.

The paper is organized as follows: in the next section, we report and discuss the main results, starting from the determination of the penetration depth and the surface resistance in zero field, to the upper critical field and main vortex pinning parameters (depinning frequency, pinning constant, vortex viscosity), to a detailed study of the flux flow resistivity. Within this study, we also compare the CPWR analysis to results obtained by means of a different microwave technique—based on the use of a dielectric-loaded resonator (DR)^6^—on similar NbTi films. Summarizing remarks and conclusions are given in the section “Conclusions and perspectives”. In the section “Methods” we report on the details of the technological processes needed to obtain the CPWR, the principles of its measurement, and details about the DR technique and the other ancillary techniques.

## Results and discussion

**Figure 1:**
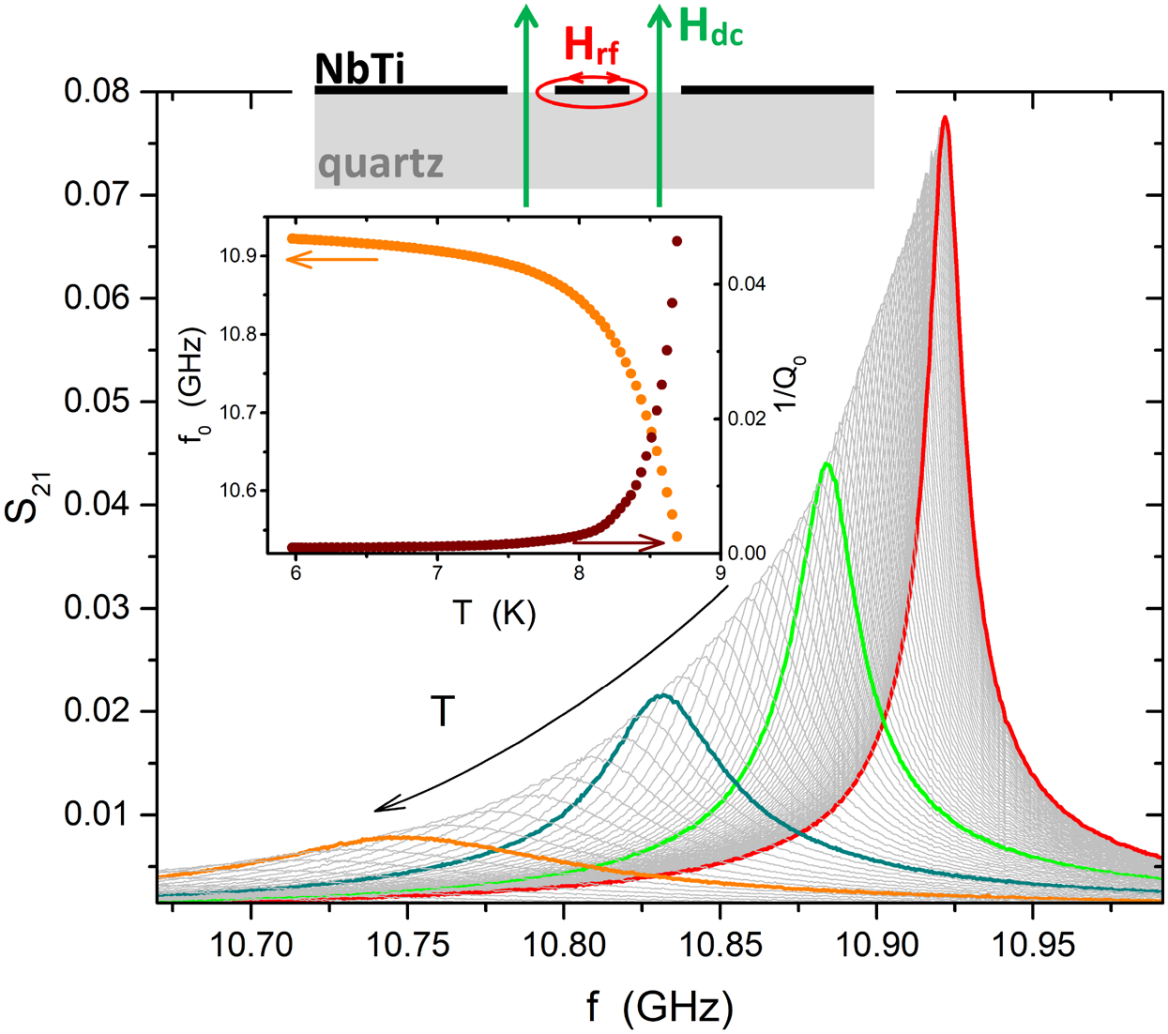
The sketch represents a section view of the CPWR (not to scale), with the NbTi lateral ground planes and central stripline (black) on the quartz substrate (gray). Red and green arrows show the directions of the rf and DC magnetic field, respectively. The main panel shows the resonance curves of the NbTi CPWR#1, measured at different temperatures, ranging from 5.9 to 8.5 K, in zero DC field. Some of the curves are highlighted, for clarity. The inset shows the temperature dependence of the resonance frequency (left scale) and the quality factor inverse (right), as deduced by a Lorentzian fit of the resonances.

CPWRs were produced by the process described in the “Methods” section. Two devices are analyzed in this work, representative of different deposition processes. In the two devices, hereafter labelled as #1 and #2, the central strip of the CPWR has the following dimensions: length-width-thickness = 8.6 mm–282 μm–1.43 μm (#1), and 8.5 mm–244 μm–2.4 μm (#2). With these geometries, the resonance frequency of both the CPWRs sets around 11 GHz. A sketch of the CPWR (not to scale), showing the directions of the rf magnetic field and of the applied DC field, is shown in Fig. 1.

### London penetration depth

Resonance curves of the CPWRs were acquired and fitted by the procedure described in the “Methods” section. Examples of curves in zero DC field and at different temperatures are shown in Fig. 1. The inset shows the temperature dependence of the fitting parameters—the resonance frequency $f_0$ and the inverse of the unloaded quality factor $Q_0$.

To obtain an estimation of the absolute value of the London penetration depth, $\lambda _L$, we investigated the resonance frequency dependence on temperature, in the absence of DC magnetic field. The resonance frequency depends on $\lambda _L(T)$ through the inductance of the line, and on the permittivity of the substrate $\varepsilon _r(T)$ through the capacitance of the line. In fact, in the theory of distributed element transmission lines for an half-wavelength resonator, $f_0$ is given by

1 $$\begin{aligned} f_0=\frac{1}{2l\sqrt{\{L_l^g+L_l^k\left[ \lambda _L\left( T\right) \right] \}C_l\left[ \varepsilon _r\left( T\right) \right] }} \end{aligned}$$

where *l* is the length of the resonator, $L_l^g$ is the geometrical inductance per unit length, $L_l^k$ is the kinetic inductance per unit length, and $C_l$ is the capacitance per unit length. The way to explicitly express $C_l$, $L_l^g$, and $L_l^k$ as a function of $\lambda _L(T)$, of the geometrical parameters of the CPWR and of the permittivity of the substrate $\varepsilon _r(T)$ is rather intricate and fully described in Refs.^7–9^. As for the temperature dependencies of $\lambda _L(T)$ and $\varepsilon _r(T)$, we adopted parametric functions allowing easy data fitting. Going into details, for $\varepsilon _r(T)$ of quartz, literature shows that at microwave frequencies it faintly depends on temperature, within the analyzed range^10^, and that its absolute values range from 4.4 to 4.6, depending on the orientation. For the sake of generality, in the fit we assume a second-order polynomial temperature dependence for $\varepsilon _r(T)$, even though we expect small linear and quadratic coefficients. Then, the way it enters in Eq. (1) depends on the geometry of the CPWR^11^.

The expression to be used for $\lambda _L(T)$ needs a deeper insight. With the aim to avoid forcing $\textit{a priori}$ a given trend, we used a very general function, that well adapts to different scenarios:

2 $$\begin{aligned} \lambda _L(T)=\frac{\lambda _L(0)}{\sqrt{1-\left( T/T_c \right) ^\gamma }} \end{aligned}$$

From a phenomenological point of view, with $\gamma =4$ Eq. (2) corresponds to the two fluids result, while $\gamma \approx 2$ was typically obtained for $d$-wave superconductors (e.g. YBa$_2$Cu$_3$O$_{7-x}$^12^). On a more fundamental basis, Eq. (2) also adapts to BCS-based calculations: the weak coupling clean limit is well approximated by $\gamma =3-T/T_c$^13^ (as successfully used e.g. with MgB$_2$^11^). However, Nb, Nb$_3$Sn and other Nb-based alloys are better described within the strong coupling regime. Curiously, this is well approximated by the phenomenological two fluids formula, with $\gamma$ slightly lower than 4 for the clean case and $\gamma$ slightly larger than 4 for the dirty case^14^. Therefore, in data fitting we adopted Eq. (2), with $\gamma$ as a fitting parameter.

**Figure 2:**
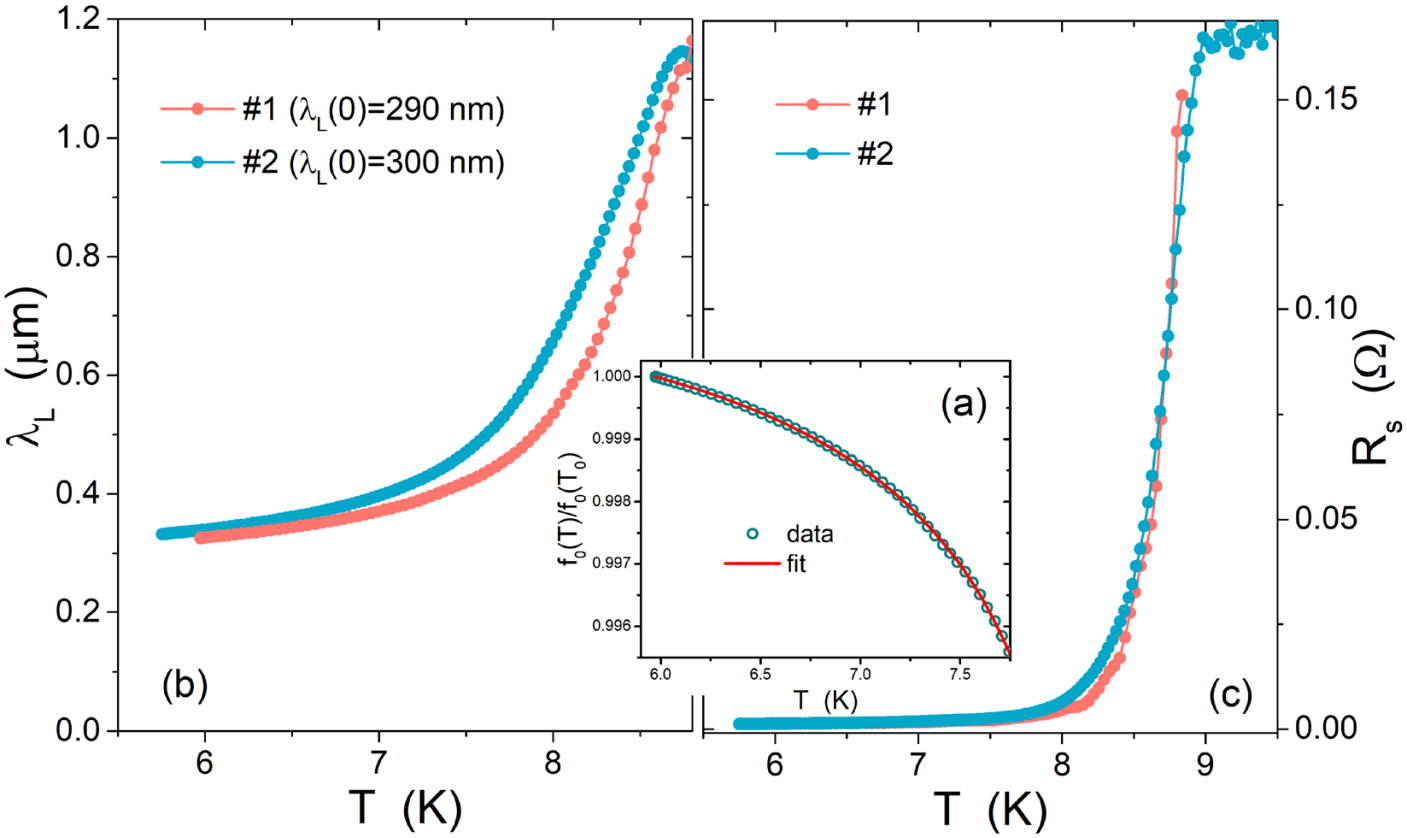
(**a**) Resonance frequency normalized to its value at the lowest measured temperature, $T_0$, as a function of temperature. Data were interpolated by means of the equations described in the text. (**b**) Penetration depth and (**c**) surface resistance, as deduced from the fitting procedure for the CPWRs #1 and #2, from measurements in zero DC field.

For the practical fitting procedure, we operated with the normalized resonance frequency that, through Eq. (1), can be written as:

$$\begin{aligned} \frac{f_0(T)}{f_0(T_0)}=\sqrt{\frac{L_l^g+L_l^k\left[ \lambda _L\left( T_0\right) \right] }{L_l^g+L_l^k\left[ \lambda _L\left( T\right) \right] }\,\frac{C_l\left[ \varepsilon _r\left( T_0\right) \right] }{C_l\left[ \varepsilon _r\left( T\right) \right] }} \end{aligned}$$

where $T_0$ is the lowest measured temperature. Taking into account all the assumptions discussed above, we fit the $f_0(T)/f_0(T_0)$ data with $\lambda _L(0), T_c, \gamma$, and the second-order coefficients for $\varepsilon _r(T)$ as fitting parameters. Further details of this fitting procedure are reported in Ref.^9^, where the way to choose a given temperature range for the fit and then to extend the determination of $\lambda _L$ to the whole range, and the way to determine the surface resistance $R_s$ are described. An example is given in Fig. 2a for CPWR#1. Results are shown in Fig. 2b,c, in terms of $\lambda _L(T)$ and $R_s(T)$.

The $R_s(T)$ curves show a very good agreement between the two CPWRs. As for the penetration depth, it should be noted that, due to the thickness *d* of the films, the condition passes from the bulk limit at low temperatures, where $2\lambda _L<d$, to the thin film limit at higher temperatures, where $2\lambda _L>d$. In the latter case, the effective penetration length is the Pearl length, $2\lambda _L^2/d$. To take into account both these limits, we used the phenomenological interpolation expression $\lambda =\lambda _L \text {coth}(2\lambda _L^2/d)$ to analyze the response of each CPWR, with its specific thickness. Then, we extracted the London penetration depth, which is characteristic of the material and independent of the CPWR dimensions, that is the one shown in Fig. 2b: the two curves reported, corresponding to the two CPWRs, define the $\lambda _L$ uncertainty for what concerns sample-to-sample repeatability. The resulting London penetration depth for $T\rightarrow 0$, as obtained on the basis of Eq. (2), is $\lambda _L(0)=(290\pm 40) \, \text {nm}$ for CPWR #1 and $\lambda _L(0)=(300\pm 30) \, \text {nm}$ for CPWR #2, and $\gamma \approx 4.1$ for both, i.e. consistent with the strong coupling dirty case. These estimations of $\lambda _L(0)$ are in accordance with results reported in Ref.^15^ (thin films, experimental) and Ref.^16^ (theoretical calculations) for NbTi with similar Ti contents.

### Effects of a DC magnetic field

**Figure 3:**
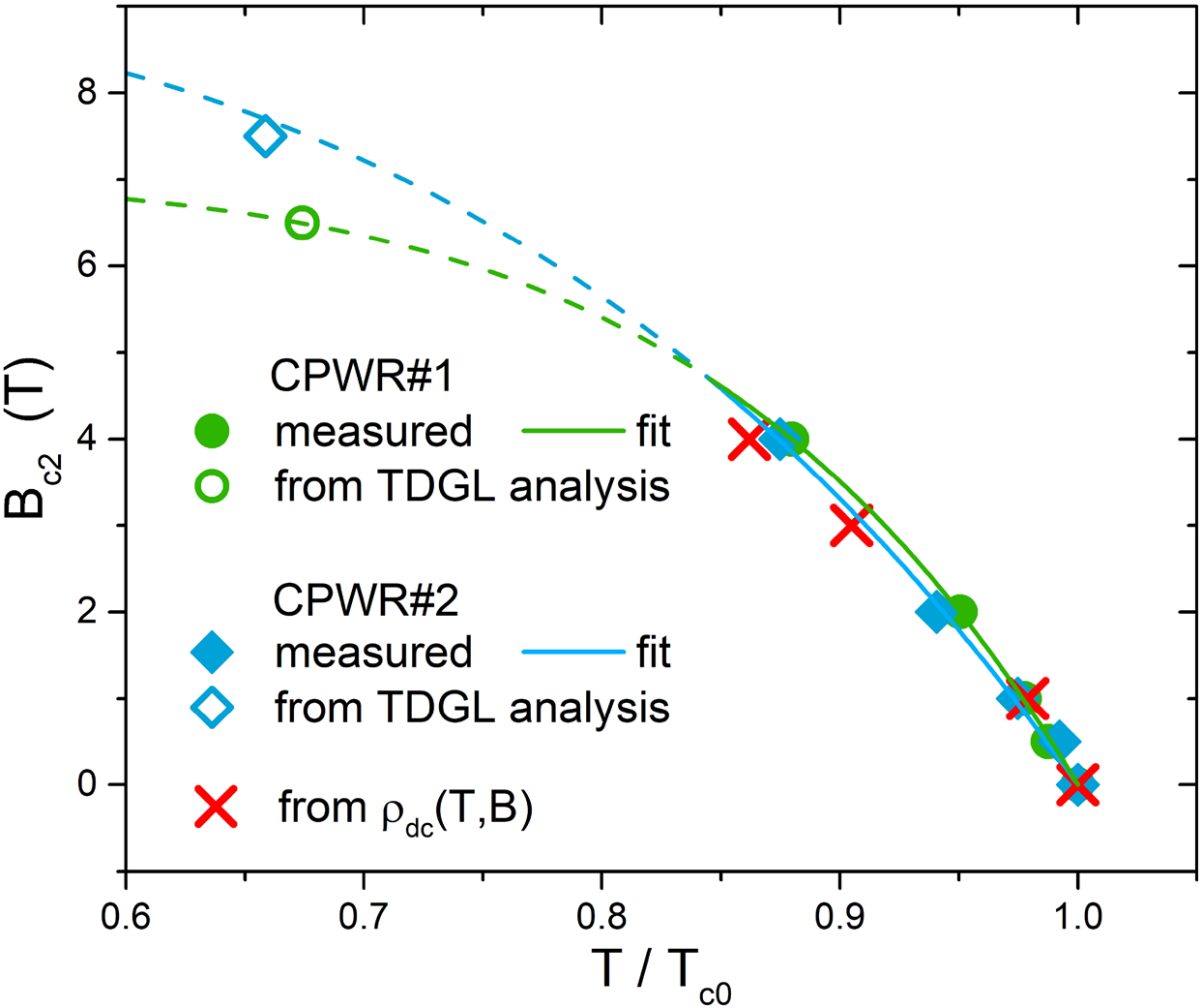
Upper critical field as a function of the reduced temperature. Solid symbols were deduced from CPWR measurements of the critical temperature for different applied DC magnetic fields, with $T_{c0}$ the critical temperature at zero field (see text). Red crosses were obtained from DC resistance measurements, by the 90% of $R_n$ criterion. Open symbols are evaluations of $B_{c2}$ at $T=6 \, \text {K}$, obtained by the TDGL analysis of $\rho _{ff}(T)$ data, as described below . Solid lines are fit of the CPWRs data (see text), extrapolated to lower temperatures (dotted lines) to check the matching with the points from TDGL analysis.

The measurements as a function of temperature were repeated for different DC magnetic fields applied perpendicular to the CPWR surface. From these measurements, the values of $T_c(H_{dc})$ can be extracted (as the onset of a superconductive response over the noise), and the upper critical field $B_{c2}(T)$ can be evaluated as the applied $\mu _0H_{dc}$ field at which $T=T_c(H_{dc})$. Figure 3 shows the data measured with both the CPWRs (solid symbols), as a function of the reduced temperature, i.e. the temperature normalized by the zero-field critical temperature $T_{c0}$. CPWR data are consistent with $B_{c2}(T)$ data obtained from DC resistance measurements *R*(*T*) of the Hall bars (red crosses), by the criterion of selecting the temperature at which the resistance reaches 90% of the normal state value, $R_n$. Note that all the curves, from the two CPWRs and from DC resistance, show a downward $B_{c2}(T)$ curvature, different from what expected by the standard Ginzburg-Landau (GL) theory. However, the curvature can still be understood within the GL theory if the granularity of the films is considered. In fact, approaching $T_c$, the coherence length $\xi _{GL}$ divergence is cut off at a typical length scale corresponding to the grain size and/or void spacing. Above this point, the $B_{c2}$ curve should assume a $(1-T/T_c)^{1/2}$ behavior^17^. Within this frame, it was also proposed that a $B_{c2}(T)$ dependence not as sharp as a square root variation can be justified by a distribution of grain sizes^17^. The presence of a granular structure supporting this hypothesis (even if with well-connected grains) is visible in our films by FESEM and can be inferred from the DC resistance normal-to-superconductor transition, with moderately large width of 0.4–0.5 K.

In Fig. 3 we show the fit (solid lines) of the CPWRs data with a generic phenomenological function $B_{c2,0}[1-(T/T_c)^\beta ]$ (values of $\beta$ of 4.1 and 6.6), extrapolated to lower temperatures (dotted lines) to check the matching with two further points (open symbols), obtained by the TDGL analysis of the $\rho _{ff}(T)$ data, as described below in the section “Flux flow resistivity”.

**Figure 4:**
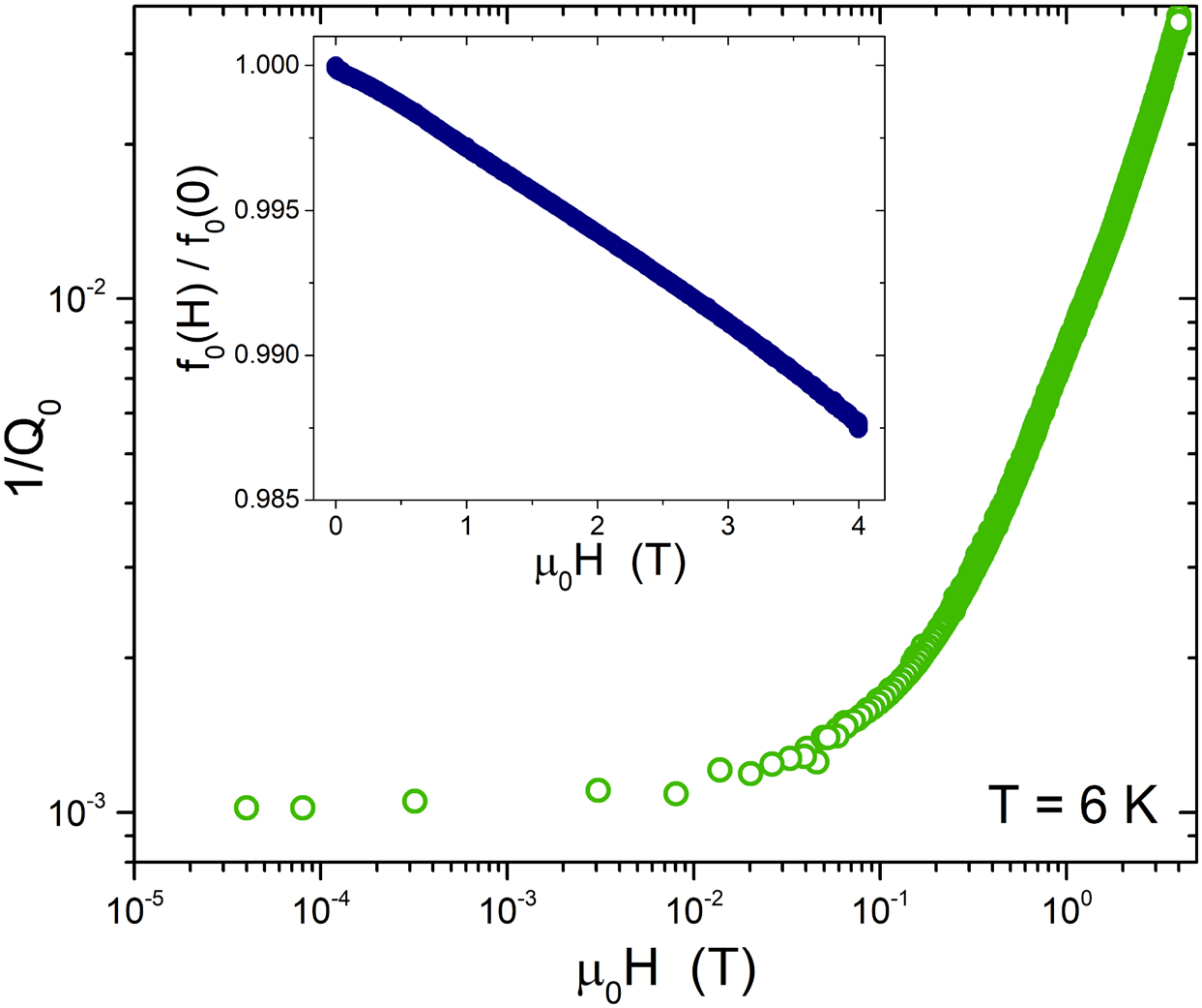
Log-log plot of the CPWR quality factor inverse as a function of the DC magnetic field, at the temperature of 6 K. The inset shows the field dependence of the normalized resonance frequency.

In Fig. 4 we report $1/Q_0$ (main panel) and the normalized $f_0$ (inset) as a function of magnetic field, at the fixed temperature of $T=6\,\textrm{K}$. *Q* degradation starts at quite low applied fields, while above about 0.5 T, a linear trend for $1/Q_0(H_{dc})$ sets up. In order to understand whether this degradation can be ascribed to vortex motion, the field of first vortex penetration should be evaluated. Actually, while $\lambda _L(0)$ is expected to increase with the Ti amount, the coherence length decreases: according to Ref.^18^, a reasonable estimation for our composition should be $\xi \approx 4.5 \, \text {nm}$. This gives a lower critical field at $T=6\,\text {K}$ of $\mu _0H_{c1}(6\,\text {K})=\Phi _0[ \text {ln}(\kappa )+1/2]/\left( 4\pi \lambda _L^2\right) \approx 6.9 \, \text {mT}$, where $\Phi _0$ is the flux quantum and $\kappa =\lambda /\xi$ is the Ginzburg-Landau parameter. The applied perpendicular field at which the first vortices are expected to penetrate the CPWR is^19^ $H_p=H_{c1}\sqrt{d/w}$ (where *d* is the thickness and *w* the width of the stripline) which for our CPWRs at $T=6\,\text {K}$ is about 0.4 mT. This value is higher than the amplitude of the microwave field, but it turns out that all the resonances whose parameters are reported in Fig. 4, with the exception of the first three points, were measured for $H_{dc}>H_p$, i.e. in the presence of vortices.

Once we established that a source of dissipation and *Q* degradation for our CPWRs can be ascribed to the presence of vortices, and more precisely to vortex dynamics, we should also consider the existence of vortex-pinning forces that act to hamper vortex motion. The main pinning parameters can be deduced by our measurements, as follows.

### Vortex-motion complex resistivity and pinning parameters

The microwave behavior of a superconductor can be described through a complex resistivity $\rho =1/(\sigma _1-i\sigma _2)$, where $\sigma _1$ is the quasiparticle conductivity and $\sigma _2=1/(\mu _0\omega \lambda _L^2)$. When vortices penetrate the sample in the mixed state, an additional complex term accounting for vortex motion, $\rho _{vm}=\rho _{vm,1}+i\rho _{vm,2}$, should be added, and the effective complex resistivity reads

$$\begin{aligned} \tilde{\rho }=\rho +\rho _{vm}=\left( \frac{\sigma _1}{\sigma _1^2+\sigma _2^2}+\rho _{vm,1}\right) +i\left( \frac{\sigma _2}{\sigma _1^2+\sigma _2^2}+\rho _{vm,2}\right) \end{aligned}$$

If $\sigma _2^2\gg \sigma _1^2$, i.e. far from $T_c$, it becomes

$$\begin{aligned} \tilde{\rho }\approx \mu _0^2\omega ^2\lambda _L^4(\sigma _1+\sigma _C)+i\mu _0\omega (\lambda _L^2+\lambda _C^2) \end{aligned}$$

with

3 $$\begin{aligned} {\begin{matrix} \lambda _C&=\sqrt{\rho _{vm,2}/(\mu _0\omega )}\\ \sigma _C&=\rho _{vm,1}/(\mu _0^2\omega ^2\lambda _L^4) \end{matrix}} \end{aligned}$$

where $\lambda _C$ is the Campbell penetration depth. The connection to the measured quantities is obtained through the surface impedance $Z_s$, that in the local limit is defined as^20^

4 $$\begin{aligned} Z_s=R_s+iX_s=\sqrt{i\mu _0\omega \tilde{\rho }} \end{aligned}$$

**Figure 5:**
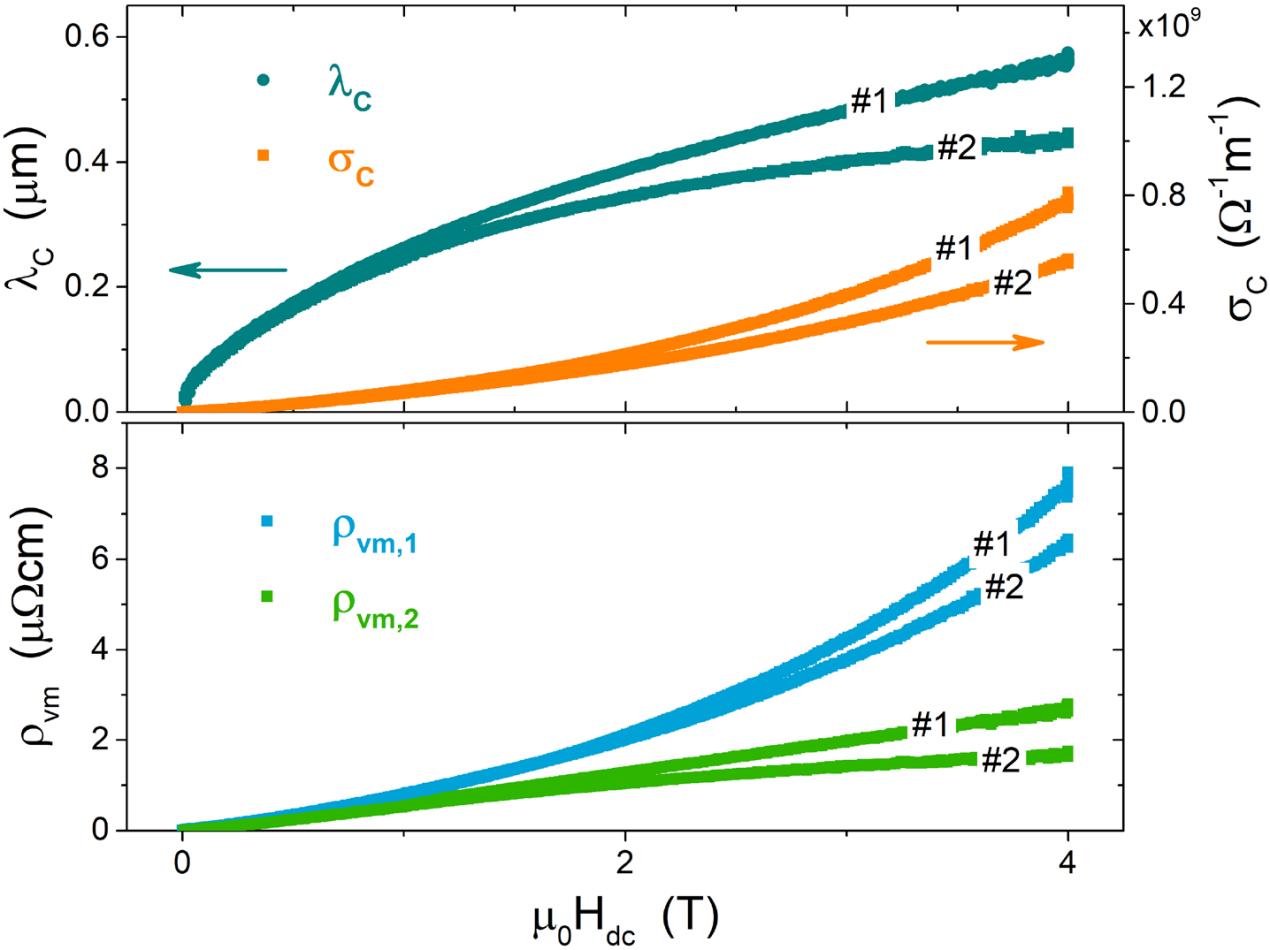
The upper panel shows the Campbell length $\lambda _C$ (left scale) and conductivity $\sigma _C$ (right scale) as a function of magnetic field, for the two CPWRs #1 and #2. The lower panel reports the real and imaginary parts of the complex resistivity due to vortex motion.

In the CPWR geometry, the surface resistance and reactance can be expresses as^8^

5 $$\begin{aligned} R_s=\omega L_l w_{eff}/Q_0 ; X_s=\omega L_l^k w_{eff}=\mu _0\omega \lambda , \end{aligned}$$

where $w_{eff}=w_{eff}[\lambda (T,H_{dc})]$ is the effective width of the strip^8,9^, and $\lambda (T,H_{dc})$ is the effective penetration depth, directly determined from the field dependence of the resonance frequency. In fact, as implicitly contained in Eq. (5), it turns out that

$$\begin{aligned} \lambda (H_{dc})=\frac{L_l^k(H_{dc})w_{eff}(H_{dc})}{\mu _0}=\frac{w_{eff}}{\mu _0} \, \left[ \frac{L_l^g+L_l^k(0)}{(f_0(H_{dc})/f_0(0))^2}-L_l^g \right] \end{aligned}$$

where the last step was derived from Eq. (1). Now, if the expression for $\tilde{\rho }$ (Eq. 3) is inserted into Eq. (4), and Eq. (5) are taken into account, one can derive the magnetic field dependence of the Campbell parameters:

6 $$\begin{aligned} \lambda _C(H_{dc})=\sqrt{\lambda ^2(H_{dc})-\lambda _L^2-(R_s^2(H_{dc})-R_s^2(0))/(\mu _0\omega )^2} \end{aligned}$$

7 $$\begin{aligned} \sigma _C(H_{dc})=\frac{L_l(H_{dc})w_{eff}[\lambda (H_{dc})]\lambda _{eff}(H_{dc})}{\pi \mu _0^2 f_0(H_{dc})Q_0(H_{dc})\lambda _L^4}-\sigma _1 \end{aligned}$$

where zero-field measurements yield $\lambda _L$ and $\sigma _1$, according to the procedure described above. Results for $\lambda _C$ and $\sigma _C$ in the case of NbTi CPWR at $T=6\,\textrm{K}$ are shown in Fig. 5. The field dependence of $\lambda _C$ is sublinear, as reported in literature for conventional superconductors^21^.

In order to extract the pinning parameters, the Gittleman-Rosenblum (GR) model is considered, connecting the vortex motion resistivity, as a function of frequency $\nu$, to the viscous drag coefficient $\eta$ of vortices, their depinning frequency $\nu _p$, and the flux-flow resistivity $\rho _{ff}$^22^:

8 $$\begin{aligned} \rho _{vm}=\rho _{vm,1}+i\rho _{vm,2}=\frac{\Phi _0B}{\eta }\frac{1}{1-i\nu _p/\nu }=\rho _{ff}\frac{1}{1-i\nu _p/\nu }. \end{aligned}$$

Note that the GR model is in principle valid only in the absence of flux creep. Thus, a proper analysis is needed to exclude significant creep. This was done by means of the dual-frequency DR measurement technique^23,24^. The measurements and corresponding analysis, briefly summarized in the “Methods” section and fully described in a forthcoming paper, allowed us to determine that a relevant role of flux creep can be ruled out in the measured NbTi samples.

Then, before using the GR model—that was initially formulated for nearly straight perpendicular vortices in thin films—one should also check whether the whole vortices or at least significant segments of them are driven by the rf field, otherwise the surface impedance could mainly be controlled by the bending rigidity of the vortex lattice. In the presently explored regime $H/H_{c1}\gg 1$, i.e. the vortex density is relatively high and uniform, so that the actual penetration depth of rf fields can be evaluated as $\Re (\tilde{\lambda })$, with $\tilde{\lambda }$ as defined by Coffey and Clem^25^. This length must be compared to the half-thickness of the film, since in CPWRs the rf field penetration occurs from both the surfaces of the film. We obtained $\Re (\tilde{\lambda })= 0.51 \, \upmu \text {m}$ and $0.97 \, \upmu \text {m}$ at the fields of 1 T and 4 T, respectively, for CPWR#1 ($0.5 \, \upmu \text {m}$ and $0.83 \, \upmu \text {m}$ for CPWR#2), to be compared with the half-thickness of $0.7 \, \upmu \text {m}$ ($1.2 \, \upmu \text {m}$). Thus, the entire vortex lines or the most part of them are effectively driven by rf currents. Moreover, we made measurements in a regime of low rf current densities, i.e. about 3000 A/cm$^2$ at 6 K and 1 T. This means that typical displacements during vortex oscillations are $\lesssim 0.1 \, \text {nm}$. In such regime, all the pinning centers, both strong and weak, are active along the whole vortex line, and bending effects are reduced. We thus conclude that the GR model is fully applicable in this case.

Starting from Eqs. (3) and (8), the parameters $\nu _p$ and $\eta$ can be expressed as a function of $\lambda _C$ and $\sigma _C$, as

9 $$\begin{aligned} \nu _p=\frac{\lambda _C^2}{\sigma _C2\pi \mu _0\lambda _L^4} \end{aligned}$$

10 $$\begin{aligned} \eta =\frac{\Phi _0B}{\mu _0^24\pi ^2\nu ^2\sigma _C\lambda _L^4\left( 1 +\frac{\lambda _C^4}{\mu _0^24\pi ^2\nu ^2\lambda _L^8\sigma _C^2}\right) } \end{aligned}$$

Finally, according to the GR model, the pinning constant $k_p$ can be calculated as

11 $$\begin{aligned} k_p=2\pi \nu _p\eta . \end{aligned}$$

**Figure 6:**
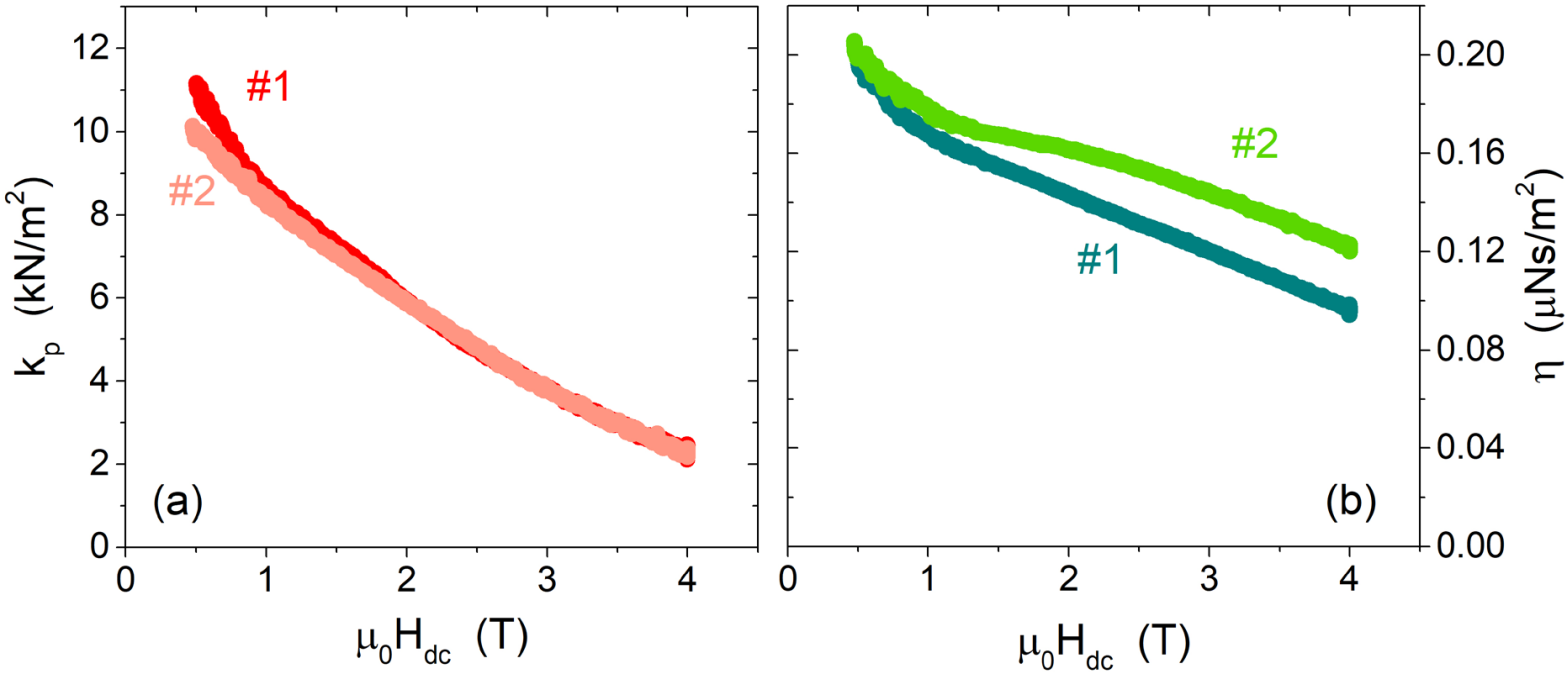
Pinning constant (**a**) and vortex viscosity (**b**) as a function of DC magnetic field at $T=6\,\text {K}$, for the two CPWRs #1 and #2.

The $k_p$ constant, characterizing the pinning force strength, and the vortex viscosity, depending on the relaxation rate of quasiparticles inside the vortex core, are shown in Fig. 6. The reported values indicate rather strong pinning, suggesting that besides grain boundaries, pinning could benefit from the possible presence of smaller and densely distributed defects (e.g. Ti precipitates). In fact, it is known that in samples with Ti concentrations exceeding 45 wt% (61 at.%), as for our CPWR samples (69 at% Ti), the main flux pinning source is from normal conducting $\alpha$-Ti precipitates^26,27^. Incidentally, the presence of Ti precipitates could also explain other peculiarities, i.e. the measured values of normal state resistivity and upper critical field smaller than values reported in literature for the nominal composition, since the presence of Ti precipitates would imply a lower Ti content in the alloy itself, and thus an actual composition different from that overall measured by EDX on a macroscopic area. Figure 6 also shows virtually absent (a) or moderate (b) sample-to-sample variability.

**Figure 7:**
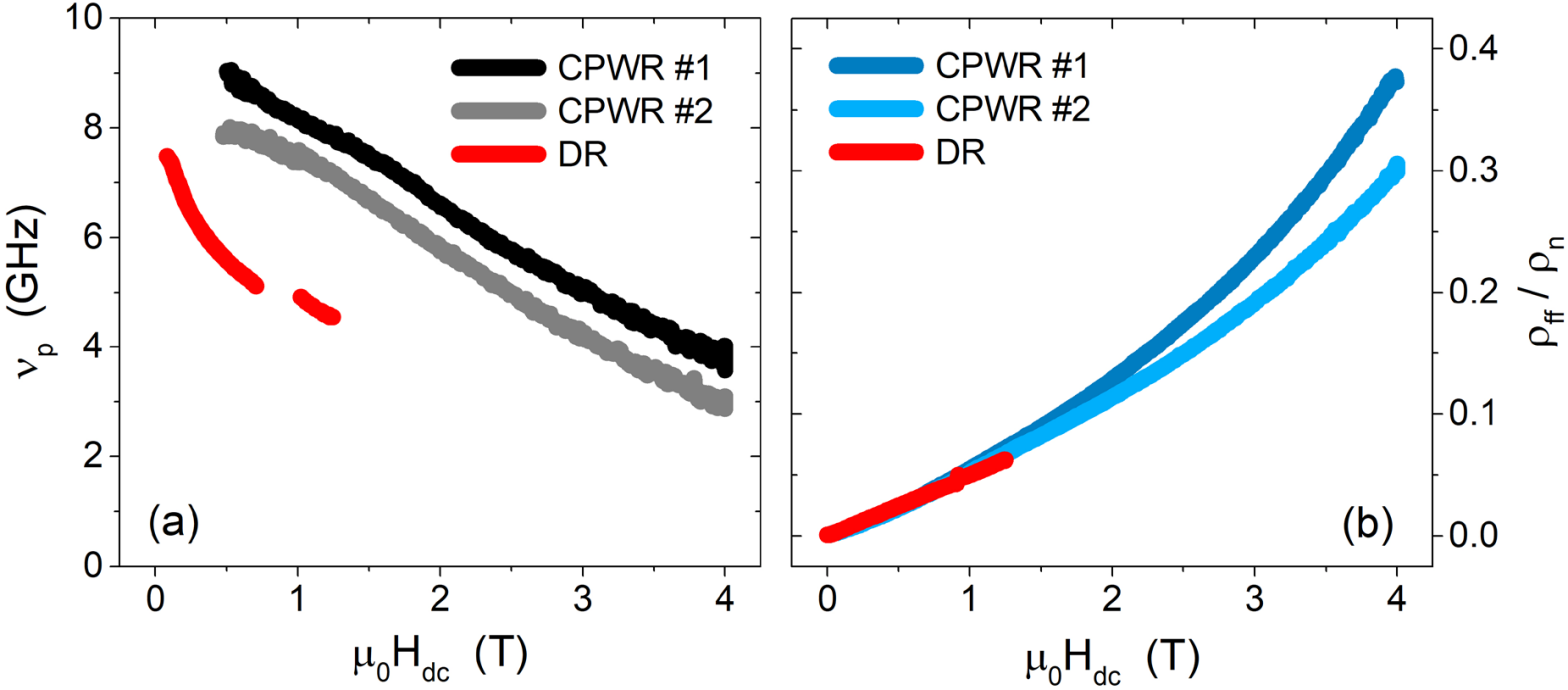
Depinning frequency (**a**) and normalized flux flow resistivity (**b**) as a function of DC magnetic field. Data from the two CPWRs #1 and #2 are compared to data from the DR analysis.

In the comparison with the DR data, one should take into account that DR measurements were done on NbTi films with a different nominal composition (62.3 at% Ti, i.e. lower than CPWR samples and at the limit for the formation of Ti precipitates) and therefore they could present differences in their pinning properties, as well as they actually show different normal-state resistivity values. In Fig. 7a we report the depinning frequency $\nu _p$ for the two CPWRs (again showing reasonable sample-to-sample variability), compared to the results of the DR measurements. DR data show lower $\nu _p$, reflecting a lower pinning force, even by including the different values of the normal state resistivities, and this is consistent with the hypothesis that precipitation of normal conducting $\alpha$-Ti could be negligible for this Ti concentration. Nevertheless, the order of magnitude of $\nu _p$ is the same for CPWR and DR, and also the slope of the field dependence is very similar, suggesting the existence of a collective pinning regime in all the samples. Where CPWR and DR data collapse is in the scaling of the flux flow resistivity, shown in Fig. 7b. In fact, despite a significant difference in the normal state properties, the curves of the flux flow resistivity $\rho _{ff}$ normalized to the normal state resistivity $\rho _n$ clearly overlap. A deeper analysis of the flux flow resistivity is presented in the next section.

### Flux flow resistivity

**Figure 8:**
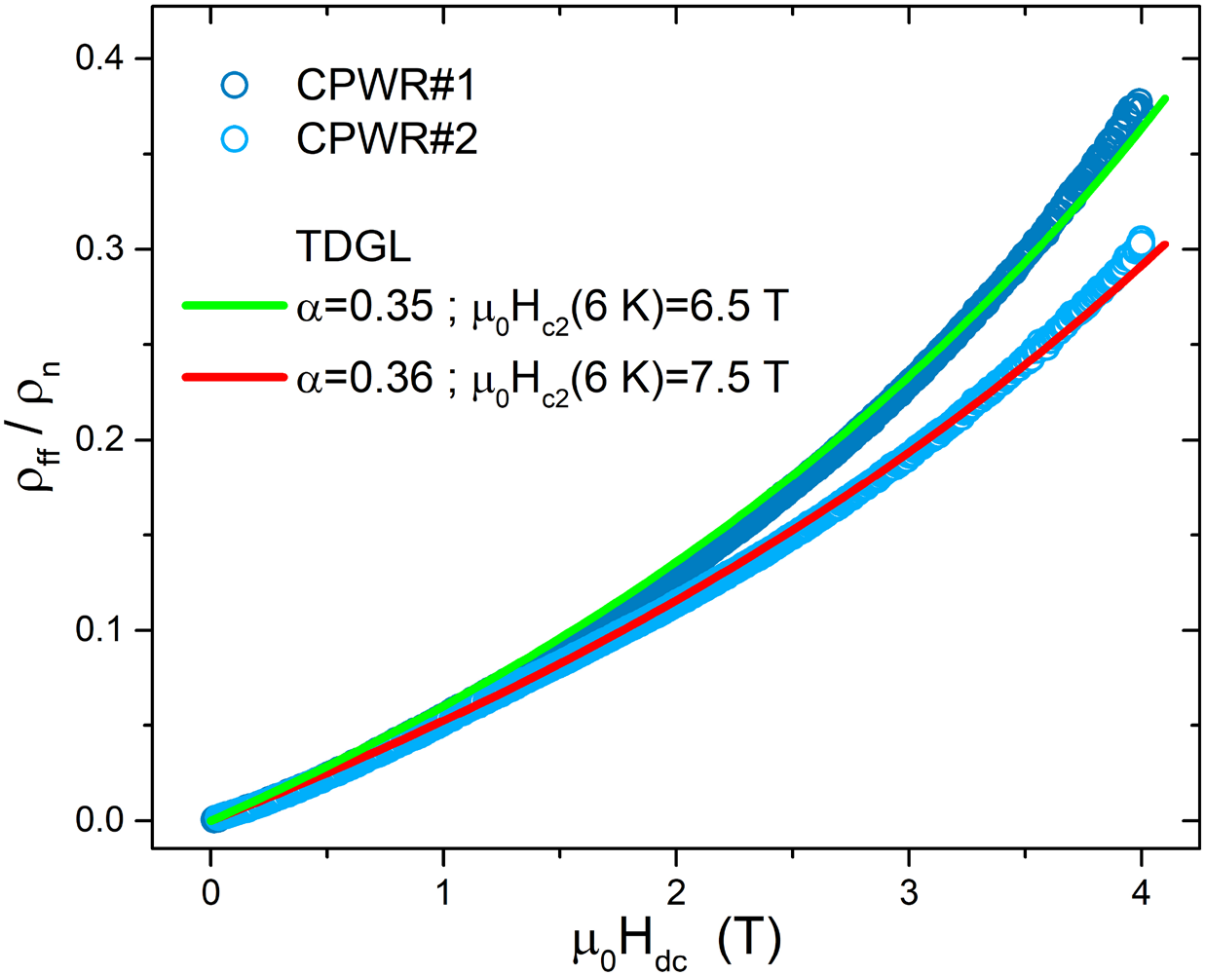
Flux flow resistivity normalized to the normal state resistivity, as a function of the applied magnetic field. Experimental data from CPWRs #1 and #2 are reported as blue symbols. The green and red lines represent the time dependent Ginzburg-Landau trends (TDGL, see Eq. 12), with the $\alpha$ and $\mu _0H_{c2}(6 \, \text {K})$ values reported in the legend.

Figure 8 reproposes the flux-flow resistivity normalized to the normal-state resistivity $\rho _{ff}/\rho _n$ as a function of the applied magnetic field, for the data collected at the fixed temperature of $T=6 \, \text {K}$. The $\rho _n(T_c)=22.5 \, \mu \Omega \, \text {cm}$ value was determined from the four-contact DC resistivity measurements of an Hall bar from the same NbTi films, as described in the “Methods” section. As a first attempt, this plot should be discussed in the framework of the Bardeen-Stephen (BS) model^28^. According to BS, in dirty s-wave superconductors the dissipation due to vortex motion is to be ascribed to the quasiparticle currents flowing in the normal cores of vortices, resulting in $\rho _{ff}/\rho _n=B/B_{c2}$. Clearly our data do not follow this trend, since they show an upward curvature. The reasons for this behavior must be found beyond the elementary theories of flux flow. Deviations from the simple proportionality $\rho _{ff}\propto B$ (i.e. $\rho _{ff}$ simply proportional to the density of vortices) emerge when the suppression of the order parameter with increasing field and modifications in the current patterns around vortices are considered. Among the most convincing approaches, the time dependent Ginzburg-Landau (TDGL) theory can be used to determine the detailed $\rho _{ff}(T,B)$ behavior beyond the BS approximation. According to the applicable mean-field result^29^,

12 $$\begin{aligned} \frac{\rho _{ff}}{\rho _n}=\frac{\alpha B}{(\alpha -1)B+\mu _0H_{c2}}. \end{aligned}$$

To the first order $\alpha \approx 0.4$, remarkably independent of temperature, field, and material parameters. The field dependence is about linear only when $B\ll \mu _0H_{c2}$, where $\rho _{ff}/\rho _n\simeq \alpha B/B_{c2}$, but even in this case the BS result is not recovered since $\alpha <1$. At increasing *B* an upward curvature is expected.

For each CPWR we found the $B_{c2}(6\, \text {K})$ value that adapts the Eq. (12) curve to the experimental data, resulting in $\alpha$ values close to the expected 0.4. A rather good agreement was found for $\alpha =0.35{-}0.36$ for the two CPWRs. Moreover, the $B_{c2}(6\, \text {K})$ values obtained by this method were plotted above in Fig. 3, to show their consistency with the $B_{c2}$ values evaluated from direct measurements at higher temperatures.

Comparing CPWR with DR-measured samples, the overlapping $\rho _{ff}/\rho _{n}$ reported in Fig. 7b implies that the ratios $\alpha /B_{c2}$ in all the samples (in the different field range explored) are essentially the same. Assuming the expected universal value $\alpha =0.4$ also for the DR sample, this would imply compatible values of $B_{c2}$ between the CPWR and DR samples also considering the uncertainty in the trend extrapolation to higher fields for the DR measurements, more limited in terms of the maximum $H_{dc}\le 1.2\;$T applied.

Overall, the flux flow resistivity analysis shows that data can be understood within the frame of the TDGL theory. In fact, a TDGL model approach was recently used to explain the vortex-induced nonlinear rf response of Nb SRF cavities^30,31^.

## Conclusions and perspectives

The adopted coplanar-waveguide-resonator technique revealed to be reliable to study the high-frequency properties of superconducting films in the presence of DC magnetic fields. By analyzing a complete set of measurements, we were able to obtain the absolute value of the penetration depth, the complex impedance and the contribution of the vortex motion to the complex resistivity, that was one of the main targets of the work. Moreover, to access the pinning parameters we analyzed the complex impedance within the formalism of the Campbell penetration depth. The complete parameter set shown in Figs. 6 and 7 comprehensively characterizes the vortex-pinning properties of the material, with moderate sample-to-sample variability.

Measurements in the GHz frequency range offer the advantage to investigate both the pinning properties and the flux flow resistivity. The last quantity was discussed and general scaling laws with normalized field were investigated. It turns out that the normalized flux flow resistivity follows the trend predicted by the time dependent Ginzburg-Landau theory remarkably well. This final result validates the whole CPWR measurement and analysis process, thus promoting the CPWR-based approach as a promising technique for the study of superconducting films in view of applications in high frequency and high field conditions.

One of the merits of the adopted approach is that all the quantities needed for the analysis, included the normalization factors, were extracted from the measurements of the very same films and devices, thus avoiding the use of data from literature which is problematic due to the high variability of parameters with the Ti content and preparation process of the samples. In fact, we could compare the CPWR results to a different microwave analysis (based on the use of a dielectric-loaded resonator), but only for NbTi films with a different composition, even if deposited in the same sputtering apparatus. Notwithstanding differences in the pinning force, the depinning frequency shows a quite reasonable agreement, and the normalized flux flow resitivities, as deduced by the two techniques, remarkably overlap.

In conclusion, the ability to arrange the whole data-set in a consistent framework of theory and pinning models is the real added value of this work, since it defines an analysis procedure and it constitutes the base to predict the behavior of the material under the application conditions and thus to design practical devices.

In perspective, the CPWR technique offers interesting possibilities for the investigation of nonlinear effects due to vortex motion, since in this configuration (rf current peaks at the edges) the nonlinearity threshold is easily achievable even with the moderate rf power of vector network analyzers. We are currently performing these studies on NbTi films and other Nb-based compounds.

## Methods

### Thin film deposition

The deposition of the NbTi films onto quartz substrates was carried out by DC magnetron sputtering using a 4″ commercial planar source with a target-sample distance of 11 cm. We deposited two sets of films, with different experimental conditions: the chamber was baked at 600 °C for 24 h and 48 h for the two runs, giving a base pressure before the process at 550 °C of $1\cdot 10^{-7} \, \text {mbar}$ and $2\cdot 10^{-8} \, \text {mbar}$, respectively. The processes were then carried out at 550 $^\circ$C fixing the current at 1.5 A (corresponding to a current density of 0.148 A/cm$^2$ and to a voltage of about 400 V). Argon was used as working gas, with a pressure of $6\cdot 10^{-3} \, \text {mbar}$. The deposition times were 20 min in the first process and 40 min in the second one. After deposition and cooling to room temperature, the pressures of the system were $2\cdot 10^{-8} \, \text {mbar}$ and $7\cdot 10^{-10} \, \text {mbar}$, respectively. The thicknesses of the films were measured using a micro-profilometer and checked by Atomic Force Microscopy (AFM) on the devices, and resulted to be 1.4 μm for the first run samples and 2.4 μm for the second run (more similar to axion cavities coating).

### Device fabrication

Coplanar waveguide resonators were produced through a combination of classical lithography, sputtering depositions and reactive ion etching. Two devices were analyzed in this work (labelled as #1 and #2), representative of the two deposition processes described in the previous sub-section.

The patterning was obtained by means of an optical lithographic laser system with a resolution of $0.9$ μm. The photoresist used during the design patterning did not provide an adequate selectivity with the NbTi film, therefore the polymer mask was not durable enough to guarantee a complete etching of the superconductive film. In order to overcome this issue, an approach based on a hard mask was adopted, in particular depositing 120 nm of aluminium sputtered in UHV conditions ($2\cdot 10^{-8}$ mbar) through an argon plasma ($10^{-3}$ mbar) with a deposition rate of 1.3 nm/s. The Al layer in excess was removed in an acetone bath, useful for the photoresist dissolution.

In these conditions, a highly directional dry etching was performed, combining the chemical and physical resistance of the aluminum to the fluorine-based gas mixture for incompatibility, and to the plasma etching exploiting its thickness, respectively. Reactive Ion Etching relied on SF_6_ at $2\cdot 10^{-2}$ mbar, glowed in plasma through an ICP power of 800 W and RF power of 50 W, leading to an etching rate of 2 nm/s. The hard mask was then removed using a wet etching in a NaOH bath.

### Ancillary measurements

The morphology of the films was analyzed by field emission scanning electron microscopy (FESEM), showing the presence of grains with a mean size of about 200 nm. FESEM analysis was finally performed after the device fabrication, to check the high quality of the transferred pattern to the NbTi film, with particular attention to the sharpness of the edges.

The composition of the films was measured by EDX (Energy Dispersive X-ray Spectroscopy) over an area much greater than the dimension of the grains, and turned out to be Nb$_{0.31}$Ti$_{0.69}$ (atomic percentage). This composition was expected to maximize $H_{c2}$, rather then $T_c$^32^.

DC resistivity measurements were performed to find out parameters useful for the microwave analysis, such as the low-temperature normal state resistivity. Hall bars $15 \, \upmu \text {m}$ in width and 1 mm in length were produced with the same method described above and their resistivity was measured by a standard four probe current-biased technique. To cancel the thermoelectric voltage offset and its possible time drift, the delta method was applied: each data point is the average of three voltage readings carried out alternating the polarity of the bias current^33^. In addition, to avoid sample heating, each current pulse was 200 ms long with an interval of 5 s between two measurements. It came out that the normal state resistivity at $T\gtrsim T_c$ is $\rho _n=22.5 \, \mu \Omega \, \text {cm}$, while a residual-resistivity ratio of 1.74 was found.

### CPWR microwave measurements

The CPWR was capacitively coupled to the readout circuit in a brass package in tight thermal contact with the cold finger of a closed cycle cryocooler. The complex transmission coefficient $S_{21}$ (ratio of the voltage transmitted to the incident voltage), as a function of the driving frequency, *f*, was detected by a vector network analyzer in different conditions of temperature, DC magnetic field (applied perpendicular to the film plane), and rf input power. The resonance frequency $f_0$ and the loaded quality factor $Q_L$ were obtained from a fit of the experimental magnitude of the transmission coefficient:

13 $$\begin{aligned} |S_{21}(f)|=\frac{|S_{21}^{max}|}{\sqrt{1+Q_L^2\left( \frac{f}{f_0}-\frac{f_0}{f}\right) ^2}} \end{aligned}$$

and the unloaded quality factor, $Q_0$, was then obtained accounting for the coupling coefficients. Figure 1 shows examples of such resonance curves measured at different temperatures for the NbTi CPWR #1. The temperature dependence of the resonance frequency and of the inverse of the unloaded quality factor (obtained starting from the fit of the single curves by Eq. 13), is shown in the inset. These parameters were used to determine the London penetration depth and the surface resistance, as detailed above.

### Dielectric-loaded resonator technique

Another NbTi sample was prepared to be studied, without patterning, through surface impedance measurements by means of a dielectric-loaded cylindrical resonator (DR)^6^. The sample used with the DR is $\approx 1.7$ μm thick (evaluation from calibrated deposition rate and deposition time), with 62.3 at.% Ti (46% weight), grown on a square quartz substrate, 1.2 mm thick and with an area of $25\times 25$ mm$^2$. The normal-state resistivity at $T\gtrsim T_c$, as obtained by the van der Pauw method with the nominal thickness, is 54 $\upmu \Omega \text {cm}$.

Following a perturbation approach, the sample was placed on one of the resonator bases and covered with a thin metal mask with a circular hole (diameter $=17\;$mm) to preserve the circular symmetry. The sample thus contributes to the resonator unloaded quality factor $Q_0$ and resonance frequency $f_0$. From the $Q_0$ and $f_0$ field induced variations, the sample surface impedance variations with $H_{dc}$ can be obtained^6^:

14 $$\begin{aligned} \Delta Z_s(H_{dc})=Z_s(H_{dc})- Z_s(0)=G_s\left[ \left( \frac{1}{Q_0(H_{dc})}-\frac{1}{Q_0(0)}\right) -2 \, \text {i} \left( \frac{f_0(H_{dc})-f_0(0)}{f_0(0)}\right) \right] \end{aligned}$$

The resonator can be excited at two electromagnetic modes (transverse electric modes TE$_{011}$ and TE$_{021}$ with resonant frequencies $f_{01}=16.5\;$ GHz and $f_{02}=26.7\;$ GHz, respectively), so that $\Delta Z_s(H)$ (and the extracted $\rho _{vm}$) is measured at the two distinct frequencies $f_{01}$ and $f_{02}$^24^. The availability of four observables (real and imaginary parts of the surface impedance at two distinct frequencies) makes possible to use more extended $\rho _{vm}$ models^23,25,34^ with respect to the GR model and to check its basic assumption about the negligibility of creep. Measurements were performed at fixed, selected temperatures between 5 K and $T_c\simeq 9.1\;$ K, by applying $H_{dc}\le 1.2\;$ T normally to the sample surface.

Similarly to what observed with the CPWR, the penetration depth of the microwave e.m. fields in the NbTi film varies, changing the regime from bulk to thin film, by increasing *T* and $H_{dc}$. The finite thickness effects are properly taken into account resorting to surface impedance transformation relations^6,20^. It can be observed that at 6 K these effects impact the vortex parameters values, with respect to the bulk limit, by $\sim 10\%$. Vortex parameters can be extracted resorting to the Coffey-Clem (CC) model^25^:

15 $$\begin{aligned} \rho _{vm}=\rho _{ff}\frac{\chi +\text {i} \nu /\nu _c}{1+\text {i} \nu /\nu _c} \end{aligned}$$

where $\chi \in [0,1]$ as an adimensional thermal creep factor and $\nu _c$ is a characteristic frequency, which reverts to $\nu _p$ for $\chi \rightarrow 0$. Indeed, the CC model expression for $\rho _{vm}$ with zero creep is equal to the GR expression, Eq. (8). The full analysis of the surface impedance data (to be published in a forthcoming paper) allowed us to assess that creep effects at low *T* (6 K included) are indeed negligible, confirming the approach taken in analysing CPWR measurements. The other two vortex parameters, $\rho _{ff}$ and $\nu _p$, are shown in Fig. 7.

## Data Availability

The datasets used and analysed for this study are available from the corresponding author on reasonable request.
